# Transition from Hepatopulmonary Syndrome to Portopulmonary Hypertension: A Case Series of 3 Patients

**DOI:** 10.1155/2013/561870

**Published:** 2013-11-10

**Authors:** Radhika Zopey, Irawan Susanto, Igor Barjaktarevic, Tisha Wang

**Affiliations:** Division of Pulmonary and Critical Care Medicine, University of California, 757 Westwood Plaza, Suite 7501, Los Angeles, CA 90024, USA

## Abstract

Hepatopulmonary syndrome (HPS) and portopulmonary hypertension (PPHTN) are the two major pulmonary vascular complications of liver disease. While HPS is characterized by low pulmonary vascular resistance, PPHTN is defined by the presence of elevated pulmonary vascular resistance. Given these seemingly opposing pathophysiologic mechanisms, these conditions were traditionally felt to be mutually exclusive. In this series, we present three patients with severe hepatopulmonary syndrome who had spontaneous resolution of their HPS with the subsequent development of PPHTN. To our knowledge, this is the largest case series presented of this phenomenon in nontransplanted patients. One proposed mechanism for the occurrence of this phenomenon involves dysregulation of the same vascular signaling pathway, which may lead to both pulmonary vascular dilatations and pulmonary arterial remodeling in the same patient. Another theory involves the possible differential binding of endothelin-1, a vasoactive signaling peptide that induces vasoconstriction when bound to receptor A and vasodilation when bound to receptor B. Although the mechanisms for this phenomenon remain unclear, it is important to be vigilant of this phenomenon as it may change the patient's overall treatment plan, especially in regard to appropriateness and timing of liver transplant.

## 1. Introduction

Hepatopulmonary syndrome (HPS) and portopulmonary hypertension (PPHTN) are two of the most common pulmonary complications of liver disease [1]. Although these two conditions may present similarily clinically and are pathologically linked by the presence of portal hypertension, their pathophysiologic mechanisms significantly differ. While HPS is characterized by low pulmonary vascular resistance secondary to intrapulmonary vascular dilatations, PPHTN features elevated pulmonary vascular resistance and constriction of the pulmonary vasculature [2]. Given their seemingly opposing pathophysiologic mechanisms, these conditions were traditionally felt to be mutually exclusive. However, there have been a few reported cases in the literature of these two conditions coexisting in patients with liver disease and one case report of spontaneous conversion from HPS to PPHTN [3]. In this series, we present three patients with severe HPS who had spontaneous resolution of their HPS with the subsequent development of PPHTN. To our knowledge, this is the largest case series published of this phenomenon in nontransplanted patients.

## 2. Case Reports

**Case 1**

**Figure 1:**
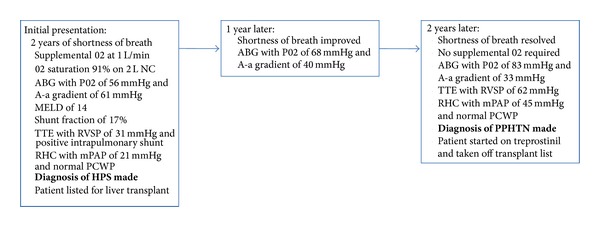
Sequence of events for Case 1.

A 57-year-old Caucasian female with a history of cirrhosis secondary to nonalcoholic steatohepatitis as well as mild asthma presented with a two-year history of shortness of breath. The patient had been placed on supplemental oxygen for significant hypoxemia approximately 1 year after the onset of her shortness of breath. At the time of her presentation to our center, the patient was requiring 1 liter of supplemental oxygen per minute at rest and 3 liters per minute (L/min) with exertion. Her asthma was not felt to play a role in her dyspnea as it had been well controlled off bronchodilators for a period of greater than 10 years. Physical exam was notable for an oxygen saturation of 91% on 2 L/min of oxygen and mild digital clubbing. Her Model for End-Stage Liver Disease (MELD) score at the time of presentation to our center was 14. The patient's computed tomography (CT) scan of the chest showed areas of mild ground glass opacities in the right upper lobe with adjacent pleural thickening and no evidence of thromboembolic disease. A wedge lung biopsy had previously been performed and the pathology had returned without evidence of interstitial fibrosis, vascular dilatations, intimal thickening, or arteriovenous malformations but did show nonspecific focal areas of eosinophilic vasculitis. Contrast-enhanced echocardiogram showed an estimated right ventricular systolic pressure (RVSP) of 31 to 36 mmHg and evidence of an intrapulmonary shunt. Pulmonary function tests (PFTs) showed an isolated impairment in diffusing capacity (DLCO) of 31% predicted. The patient also underwent a technetium 99-macroaggregate albumin scan that revealed a 17% right to left shunt. An arterial blood gas (ABG) on room air revealed a pH of 7.46, partial pressure of oxygen in the arterial blood (PO2) of 56 millimeters of mercury (mmHg), partial pressure of carbon dioxide in the arterial blood (PCO2) of 26 mmHg, and an alveolar-arterial (A-a) gradient of 61 mmHg. These findings were consistent with a diagnosis of hepatopulmonary syndrome and the patient was placed on the liver transplant (LT) list with a MELD exception for HPS. Prior to listing, the patient underwent a surveillance right heart catheterization (RHC) showing a mean pulmonary artery pressure (mPAP) of 21 mmHg and a normal pulmonary capillary wedge pressure (PCWP). Over the next three years, the patient's shortness of breath and hypoxia gradually improved and she was slowly weaned off of oxygen (Figure 1). No specific therapy was given over this period of time for her HPS. During this time period, her liver disease remained stable without any treatment and her MELD score improved from 14 to 11. An ABG on room air two years later showed a pH of 7.44, PO2 of 68 mmHg, PCO2 of 33 mmHg, and A-a gradient of 40 mmHg. A subsequent ABG four years later showed a pH of 7.48, PO2 of 83 mmHg, PCO2 of 27 mmHg, and A-a gradient of 33 mmHg. A routine transthoracic echocardiogram (TTE) performed two years later, however, showed an elevated RVSP of 62 mmHg, suggestive of pulmonary hypertension. She therefore underwent repeat RHC, which showed a markedly elevated mPAP of 45 mmHg, a cardiac output (CO) of 5.5 L/min, a pulmonary vascular resistance (PVR) of 372 dynes s^−1 ^cm^−5^, and a PCWP of 12 mmHg, consistent with pulmonary arterial hypertension. A rheumatologic workup was unrevealing and the patient was subsequently considered for treatment with treprostinil, a prostacyclin analog. She was taken off the liver transplant list until successful treatment of her PPHTN and her care was transferred back to the referring center.

**Case 2**

**Figure 2:**
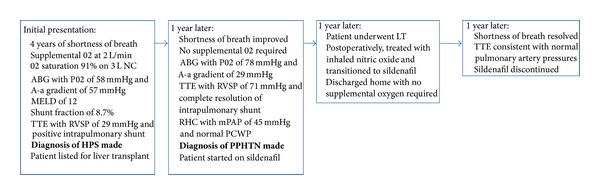
Sequence of events for Case 2.

A 63-year-old Caucasian male with hepatitis C cirrhosis presented with a 4-year history of worsening dyspnea on exertion. He reported a history of platypnea and his symptoms had progressed to severe dyspnea with any activity. Approximately 1 year prior to presentation, the patient had been admitted to an outside hospital for hypoxemia and had been placed on supplemental oxygen. At the time of presentation to our center, the patient was using supplemental oxygen at 2 L/min at rest and 5-6 L/min with exertion. On physical exam, the patient's oxygen saturation was 90% on 3 L/min of oxygen and he had marked digital clubbing and peripheral cyanosis. The patient's MELD score at the time of presentation was 12. The patient's CT scan of the chest showed mild peripheral pulmonary fibrosis and enlargement of the distal pulmonary arterioles and nontapering pulmonary vessels, suggestive of hepatopulmonary syndrome. Contrast-enhanced echocardiography revealed an RVSP of 29 mmHg and an intrapulmonary shunt. The patient's pulmonary function tests demonstrated an isolated severe impairment in DLCO of 31% predicted. An ABG on room air revealed a pH of 7.45, PO2 of 58 mmHg, PCO2 of 28 mmHg, and A-a gradient of 57 mmHg. An oxygen shunt study with 100% oxygen given via face mask also revealed an elevated shunt fraction of 8.7%. Given these findings, the patient was diagnosed with severe HPS and listed for LT with a MELD exception. One year after his initial evaluation, the patient's shortness of breath had improved and he no longer required supplemental oxygen (Figure 2). A repeat ABG showed significant improvement with pH of 7.41, PO2 of 78 mmHg, PCO2 of 34 mmHg, and A-a gradient of 29 mmHg. He was treated with supplemental garlic at 1 gram twice daily for HPS over that period of time. Repeat imaging of his chest showed unchanged mild peripheral pulmonary fibrosis and he subsequently underwent a surveillance TTE one year after his initial evaluation, which showed a significantly elevated RVSP of 71 mmHg, raising concern for PPHTN. Contrast-enhanced echocardiography at the time showed complete resolution of the previously seen intrapulmonary shunt. The patient subsequently underwent RHC, which showed a markedly elevated mPAP of 40 mmHg, a CO of 7.0 L/min, a PVR of 274 dynes s^−1 ^cm^−5^, and PCWP of 16 mmHg, consistent with pulmonary arterial hypertension. The patient was started on sildenafil 20 mg three times a day. Two years after his initial evaluation for hypoxemia and one year after his diagnosis of PPHTN, the patient underwent a deceased-donor orthotopic LT. The patient's MELD score prior to transplant was 28. Perioperatively, the patient was treated with intravenous epoprostenol. After liver transplantation, the patient was started on inhaled nitric oxide and subsequently transitioned to sildenafil 100 mg three times a day. His postoperative course was complicated by abdominal bleeding requiring repeat exploratory laparotomy, renal failure necessitating dialysis, and difficulty weaning from the ventilator. Despite these complications, he was discharged home in good condition with no supplemental oxygen requirements. One year later, the patient's dyspnea on exertion almost completely resolved and TTE revealed a time to peak velocity of the right ventricular outflow tract of 126 milliseconds, which was consistent with normal pulmonary artery pressures. Given this finding and resolution of symptoms, the patient's sildenafil was discontinued.

**Case 3**

**Figure 3:**
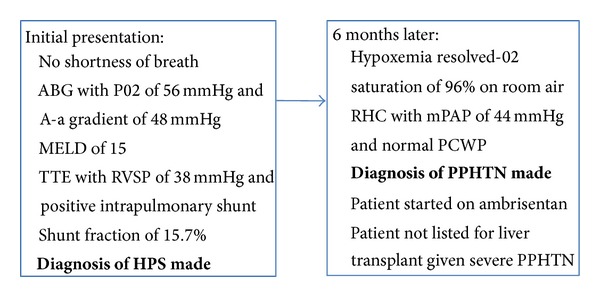
Sequence of events for Case 3.

A 56-year-old Hispanic male with hepatitis C cirrhosis and associated hepatocellular carcinoma with a MELD score of 15 was evaluated for liver transplantation. Despite a relative lack of pulmonary symptoms, routine PFTs showed a diffusing capacity of 80% of predicted. An ABG on room air revealed a pH of 7.41, PO2 of 56 mmHg, PCO2 of 37 mmHg, and A-a gradient of 48 mmHg. A 100% oxygen shunt study revealed an elevated shunt fraction of 15.7%. A CT scan of the chest demonstrated no evidence of pulmonary parenchymal disease, and a contrast-enhanced echocardiogram performed revealed a pulmonary shunt consistent with HPS and an RVSP of 38 mmHg. Six months after his initial presentation, the patient presented to our center, again without pulmonary symptoms, and his hypoxemia had resolved with a room air oxygen saturation of 96% (Figure 3). His liver disease was stable with a MELD score of 15. As part of his LT evaluation, he underwent RHC, which showed an elevated mPAP of 44 mmHg, CO of 4.9 L/min, PVR of 462 dynes s^−1 ^cm^−5^, and a PCWP of 6 mmHg, diagnostic of pulmonary arterial hypertension. Nitric oxide was given during the right heart catheterization and the patient's mean pulmonary artery pressure did decrease to 31 mmHg. Due to the patient's severe PPHTN, he could not be listed for LT. The patient was initiated on ambrisentan and his care was transferred back to the referring center, with plans to reevaluate the patient for LT once his PPHTN is better controlled.

## 3. Discussion

Two of the most common pulmonary complications of end-stage liver disease are hepatopulmonary syndrome and portopulmonary hypertension. Hepatopulmonary syndrome is defined as a triad of liver disease, an elevated A-a gradient on room air, and evidence of intrapulmonary vascular dilatation [1]. For a definitive diagnosis of HPS, patients must meet three criteria: (1) room air PO2 < 80 mmHg or A-a gradient > 15 mmHg, (2) evidence of intrapulmonary shunting (typically on contrast-enhanced echocardiography or a lung perfusion scan), and (3) portal hypertension with or without cirrhosis [1]. Portopulmonary hypertension is defined as the presence of pulmonary arterial hypertension in the setting of portal hypertension, with or without the presence of cirrhosis. Diagnostic criteria for PPHTN include (1) mean pulmonary arterial pressure ≥ 25 mmHg at rest or 30 mmHg on exercise, (2) pulmonary vascular resistance > 240 dynes s^−1 ^cm^−5^, and (3) pulmonary capillary wedge pressure < 15 mmHg [1]. While the prevalence of HPS ranges between 4% and 29%, portopulmonary hypertension is more rare, with prevalences reported between 0% and 7% [2].

Though HPS and PPHTN are clinically distinct entities with seemingly distinct pathophysiologies, they can very rarely occur simultaneously or sequentially in the same patient. The majority of cases of sequential development of PPHTN after HPS have been reported in patients who have undergone liver transplant. In a sense, patients go from being too “vasodilated” to too “vasoconstricted” after their underlying liver disease is resolved. A review of the literature has shown four cases in which pulmonary hypertension developed after liver transplantation in adult patients with preexisting hepatopulmonary syndrome [4–7]. In the earliest case report by Kaspar et al., a 33-year-old male had HPS that resolved after OLT but then developed pulmonary hypertension 14 months later [5]. The other cases reported the development of severe pulmonary hypertension months to years after liver transplantation for HPS [4, 6].

The opposite—patients with existing PPHTN and the subsequent development of HPS—has also been reported in two separate case reports, one with a short time interval of days and one with a longer time interval of two years [8, 9]. Additionally, Pham et al. reported a case of a 46-year-old female who was diagnosed with hepatopulmonary syndrome and pulmonary hypertension simultaneously which we have also seen at our center [10]. In the limited literature, there has only been one reported case of pulmonary hypertension developing after resolution of preexisting significant hepatopulmonary syndrome in an adult nontransplanted patient, similar to what we are describing in our series [3].

Why patients with portal hypertension develop both pulmonary complications of liver disease, whether simultaneously or transitioning from one to the other, remains unclear. Both PPHTN and HPS may be the result of abnormal angiogenesis of the pulmonary microcirculation induced by chronic liver disease. As compared to PPHTN which mostly involves small muscular pulmonary arteries, the vascular remodeling process in HPS involves more distal structures such as precapillary and capillary vessels. The dysregulation of the same vascular signaling pathway may lead to both pulmonary vascular dilatations and pulmonary arterial remodeling leading to PPHTN in the same patient [7, 11]. Still, as the overall incidence of PPHTN is considerably lower than that of HPS, pulmonary arteries may be relatively protected against angiogenic stimulation and genetic susceptibility may play a role in the progression of the disease [7]. Elevated portal vein pressures, a characteristic of both disorders, may also lead to increased translocation of gram-negative bacteria and endotoxins which act as inciting factors for the release of vasoactive mediators and activation of the endothelin system [12]. Another proposed theory involves differing expressions of the endothelin-1 receptor. Endothelin-1 (ET1) is a vasoactive signaling peptide that has different effects when bound to its different receptors, A and B. When bound to receptor A, endothelin-1 leads to vasoconstriction and increased pulmonary vascular resistance [13]. In contrast, as seen in rat models of HPS, binding of endothelin-1 to receptor B leads to upregulation of endogenous nitric oxide synthetase and increased nitric oxide production, resulting in pulmonary vasodilation [14, 15]. The susceptibility to ET1 may differ during the course of the disease and the overexpression of a specific receptor and differential binding of ET1 to receptor B in HPS and receptor A in PPH may be one mechanism to explain the resolution of HPS and subsequent development of PPHTN. 

In addition, one of our patients was given garlic during his HPS course because of its potential benefit [16]. In 1994, Nagae et al. hypothesized that garlic inhibits NO synthesis in macrophages, resulting in a decreased concentration of nitric oxide, therefore decreasing vasodilation [17]. However, a rat study by Ku et al. reported that garlic and its active metabolite allicin induce endothelium- and nitric oxide-dependent relaxation in rat pulmonary arteries [18]. In a clinical study showing the benefit of garlic in HPS, Abrams and Fallon conjectured that garlic results in more uniform vasodilation within the lungs, with improvements in V/Q mismatch in the apical and mid lung fields. This improvement in V/Q mismatch may ultimately decrease NO production resulting in decreased pulmonary vasodilation and the improvement of HPS and its associated hypoxemia [16]. Whether garlic therapy contributes to the subsequent development of PPHTN remains unknown at this time.

To our knowledge, this is the largest case series reported of patients with clinically significant severe HPS with subsequent resolution of their HPS and hypoxemia with conversion to significant PPHTN. In every case, it changed the patients' eligibility, status, and treatment plan prior to LT. It is extremely important to be aware of this phenomenon so that patients who have HPS are still continuously monitored for signs and symptoms of PPHTN. At our center, we continue routine screening of these patients with transthoracic echocardiograms annually. Ultimately LT has been shown to lead to resolution of both pulmonary vascular complications of liver disease but the timing is extremely crucial in both situations with LT proven to be high risk in patients with HPS and a PaO2 < 50 mmHg and in patients with PPHTN with a mPAP > 35 mmHg [19].
